# X-ray Absorption Spectroscopy Study of Thickness Effects on the Structural and Magnetic Properties of Pr_2−δ_Ni_1−x_Mn_1+x_O_6−y_ Double Perovskite Thin Films

**DOI:** 10.3390/nano12234337

**Published:** 2022-12-06

**Authors:** Mónica Bernal-Salamanca, Javier Herrero-Martín, Zorica Konstantinović, Lluis Balcells, Alberto Pomar, Benjamín Martínez, Carlos Frontera

**Affiliations:** 1 Institut de Ciència de Materials de Barcelona, ICMAB-CSIC, Campus UAB, 08193 Cerdanyola del Vallès, Spain; 2 ALBA Synchrotron Light Source, 08920 Cerdanyola del Vallès, Spain; 3 Center for Solid State Physics and New Materials, Institute of Physics Belgrade, University of Belgrade, Pregrevica 118, 11080 Belgrade, Serbia

**Keywords:** thin film, ferromagnetic double perovskite, X-ray absorption spectroscopy

## Abstract

In this work, we report a systematic study of the influence of film thickness on the structural and magnetic properties of epitaxial thin films of Pr_2−δ_Ni_1−x_Mn_1+x_O_6−y_ (PNMO) double perovskite grown on top of two different (001)-SrTiO_3_ and (001)-LaAlO_3_ substrates by RF magnetron sputtering. A strong dependence of the structural and magnetic properties on the film thickness is found. The ferromagnetic transition temperature (*T*_C_) and saturation magnetization (*M*s) are found to decrease when reducing the film thickness. In our case, the thinnest films show a loss of ferromagnetism at the film-substrate interface. In addition, the electronic structure of some characteristic PNMO samples is deeply analyzed using X-ray absorption spectroscopy (XAS) and X-ray magnetic circular dichroism (XMCD) measurements and compared with theoretical simulations. Our results show that the oxidation states of Ni and Mn ions are stabilized as Ni^2+^ and Mn^4+^, thus the ferromagnetism is mainly due to Ni^2+^-O-Mn^4+^ superexchange interactions, even in samples with poor ferromagnetic properties. XMCD results also make evident large variations on the spin and orbital contributions to the magnetic moment as the film’s thickness decreases.

## 1. Introduction

Double perovskite (DP) oxides of the R_2_NiMnO_6_ family (RNMO, where R is a rare earth element) have attracted much attention from the scientific community due to their potential interest for future technological applications. Particularly, these materials are attractive because, being ferromagnetic insulators (FM-Is), they are promising candidates for applications in spintronic devices, such as multiple state logic devices, magnetodielectric capacitors, and spin filters tunnel junctions [1,2,3,4,5]. Since FM-Is are very scarce, DPs of the RNMO family may play a relevant role in the future development of spintronics because they are among the few known FM-Is [6,7,8]. Magnetic tunnel junctions (MTJs), one of the most important spintronic devices, require high spin-polarized materials to enhance the performance of tunnel magnetoresistance (TMR), and FM-Is have the potential to increase the magnitude of TMR as spin-filtering barriers [9]. The spin filtering effect of FM-I barriers is caused by the spin-sensitive conductance induced by spin-dependent potentials in FM-Is [10,11].

Previous reports on this class of compounds (RNMO) have been mainly focused on La_2_NiMnO_6_ (LNMO) due to its stable ferromagnetic insulating phase, high Curie temperature (T_C_ ≈ 280 K), magneto-dielectric properties, spin-phonon coupling, and even catalytic properties [2,12,13,14]. Nevertheless, there are few studies available in the literature of other members of this family, either in bulk or thin film form, such as Pr_2_NiMnO_6_ (PNMO). According to the Goodenough-Kanamori rules, the magnetic ground states of RNMO systems are often expected to be ferromagnetic because of the superexchange interaction between the half-filled *e*_g_ orbitals of Ni^2+^$(d^{8},\;S=1$) and empty *e*_g_ orbitals of Mn^+4^ $\left(d^{3},\;S=3/2\right)$, which are ferromagnetic via 180° TM($e_{g}^{2}$)–O–TM$\left(e_{g}^{0}\right)$ geometry [15,16,17,18]. However, the presence of anti-site disorder with the interchange between Ni and Mn atom positions, as well as oxygen vacancies, may result in antiferromagnetic (AF) coupling due to superexchange interactions between Mn^4+^−O−Mn^4+^ and Ni^2+^−O−Ni^2^ ions [6,19,20,21]. Consequently, the physical properties of these materials are found to be very sensitive to the Ni/Mn ordering in the B-site of the DP structure.

The properties of the RNMO family show a gradual structural change as the A-site is occupied by rare-earth elements R^3+^ with a smaller ionic radius. As a result, the magnetic transition temperature, *T*_C_, decreases monotonously with decreasing the *r*_R_^3+^ radius and the octahedral tilting of MnO_6_ and NiO_6_ octahedra increases [8,18,22]. Therefore, the superexchange interaction between the Ni^2+^ and Mn^4+^ ions is affected due to the larger deviation of the Ni–O–Mn bond angle from 180°. Therefore, the crystal structure and the magnetic behavior of these materials are correlated not only to the Ni−O−Mn bond angle but also to the variation of covalency/ionicity of the Ni/Mn−O bond length [8,18,23,24]. On the other hand, it has been found that for RNMO double perovskites, one of the greatest challenges is to control the ordering of the B-site cations (Ni/Mn), which strongly affects the microstructure and physical properties of RNMO thin films. Particularly, B-site ordering can be influenced by several factors, such as the growth conditions (i.e., growth temperature, oxygen pressure, and annealing), the epitaxial strain induced by the film-substrate lattice mismatch, and film thickness. In this regard, it is noted that in comparison with the studies on the synthesis conditions and properties of LNMO compounds, investigations on PNMO thin films related to these growth parameters are still very scarce [2,6,19].

Our studies are focused on PNMO thin films, which are a less explored member of the RNMO family. The parent compounds of PNMO are PrNiO_3_ and PrMnO_3_ single perovskites, which have an orthorhombic *Pbnm* structure with Pr^3+^ occupying the A-site and Ni^3+^ and Mn^+3^ occupying the B-sites [25,26]. PrMnO_3_ and PrNiO_3_ are A-type and G-type antiferromagnetic insulators, while Pr_2_NiMnO_6_ double perovskite, with Pr^3+^ (~1.06 Å) occupying the A-site and Mn^4+^ (~0.53 Å), Ni^2+^ (~0.69 Å) occupying the B and B’ sites in A_2_BB’O_6_ with rock-salt type order, is a ferromagnetic insulator [27,28]. Studies have demonstrated that well-ordered Pr_2_NiMnO_6_ in bulk or film form may be arranged in a monoclinic *P*2_1_/n structure with Ni^2+^ and Mn^4+^ cations alternatively arranged at the B sites [8,27].

In a previous work [29], we have carried out a detailed optimization of the growth conditions (such as oxygen partial pressure and growth/annealing temperature) of PNMO thin films on top of (001)-oriented SrTiO_3_ (STO) substrates by RF magnetron sputtering technique. In our results, we have obtained high-quality double perovskite PNMO thin films with good ferromagnetic properties (i.e., T_C_ ≈ 210 K and Ms ≈ 4.5 µ_B_/f.u. at 10 K, very close to the bulk value) and insulating behavior using high oxygen pressures (350 mTorr) and high growth/annealing temperatures (800–850 °C). In order to obtain additional information on the composition of the films, we have analyzed the stoichiometry by electron probe microanalysis (EPMA). Particularly, the stoichiometry of the PNMO films has revealed that samples grown under high oxygen pressures (PO_2_ ≥ 350 mTorr) show a certain degree of Pr deficiency (not related to Pr migration as in, e.g., [30]). Cationic vacancies can have an impact on the properties of perovskite oxides [31,32]. Nevertheless, in spite of this Pr deficiency, the stoichiometry of the samples has little impact on the ferromagnetic properties. In this regard, the stoichiometry of our PNMO films has been expressed as Pr_2−δ_Ni_1−x_Mn_1+x_O_6−y_.

Taking into account these previous results, in the first part of this work, we study the dependence of the structural and magnetic properties on the film thickness of PNMO samples deposited on top of two different substrates, namely (001)-oriented SrTiO_3_ (STO) and (001)-oriented LaAlO_3_ (LAO). The purpose of selecting two types of substrates, which impose different structural strains, is to evaluate the effect of structural strain (induced by lattice mismatch with the underlying substrate) on the crystal structure of the films and its impact on the ferromagnetic properties. At the same time, the structural strain is also expected to be strongly dependent on the film thickness. Strain effects often modify both in-plane and out-of-plane lattice parameters when varying the film thickness. In this regard, examining the physical properties of ultrathin PNMO films (~3 nm thick) could be useful for applications such as spin filters in tunnel barriers. In our case, both the structural and ferromagnetic properties of PNMO films have shown strong dependence on film thickness. In particular, the thinnest films showed a loss of ferromagnetism at the interface. On the other hand, the selection of the substrates also plays an important role in controlling the nature of magnetic anisotropy. Additionally, in the second part of this paper, we have focused our attention on exploring the local electronic structure of some representative PNMO samples deposited on STO and LAO substrates (both for high and low-*T*_C_), using X-ray absorption spectroscopy (XAS) and X-ray magnetic circular dichroism (XMCD) measurements. Finally, experimental data have been compared with theoretical simulations of the XAS and XMCD spectra.

## 2. Materials and Methods

Pr_2−δ_Ni_1−x_Mn_1+x_O_6−y_ (PNMO) films were deposited on top of (001)-oriented SrTiO_3_ (STO) and (001)-oriented LaAlO_3_ (LAO) substrates by RF magnetron sputtering technique, using a stoichiometric Pr_2_NiMnO_6_ target prepared by the solid-state reaction method [29]. Before deposition, substrates were cleaned in an ultrasonic bath with Milli-Q water and then annealed at 1000 °C in air for 2 h to obtain a clean and smooth step-terrace morphology [33,34].

Films were grown under an oxygen pressure of 350 mTorr and a growth temperature of 800 °C. The optimization of the growth conditions and the stoichiometry of the samples (obtained by EPMA) have been reported elsewhere [29]. The film thickness (t) determined by X-ray reflectivity was modulated by varying the deposition time (i.e., 60, 30, 15, 7, 5, and 3 min). After thin film growth, samples were annealed in-situ at the same growth temperature (800 °C) for 1 h under high oxygen pressure (420 Torr) and then slowly cooled down to room temperature at 10 °C/min. All PNMO films were prepared at a fixed RF power of 40 W and a fixed target-to-substrate distance of 5 cm, respectively.

The surface morphology of the samples was characterized by atomic force microscopy (AFM, MFP-3D AFM Asylum Research, Goleta, CA, USA) in tapping mode. The crystallinity quality of the samples was studied by X-ray diffraction (XRD), and the film thickness was determined by X-ray reflectivity (XRR) using a Bruker D8-Advance and a Siemens D5000 diffractometer (Cu-K_α1_ and Cu-K_α1,2_ radiation, respectively, both from ICMAB’s scientific and technical services). Synchrotron X-ray diffraction measurements were performed using the KMC-II beamline of BESSY (Berliner Elektronen-Speicherring Gesellschaft für Synchrotronstrahlung, Hemholtz Zentrum Berlin). Magnetization measurements were done using a superconducting quantum interferometer device (SQUID, Quantum Design, from ICMAB’s scientific and technical services).

X-ray absorption spectroscopy (XAS) and X-ray magnetic circular dichroism (XMCD) were investigated at the Pr *M*_4,5_, Ni *L*_2,3,_ Mn *L*_2,3_ and O *K* edges in the BL29-BOREAS beamline [35] at the ALBA Synchrotron Light Source (Barcelona, Spain). The spectra were measured in total electron yield (TEY) mode at T = 100 K under ultrahigh vacuum conditions (2 × 10^−10^ mbar). The applied magnetic field (parallel to the X-ray beam) was 2 T. These experiments were also supported by theoretical simulations. The degree of circular polarization of the beam in the energy range used is higher than 99% [35].

## 3. Results and Discussion

### 3.1. Structural Properties

**Figure 1:**
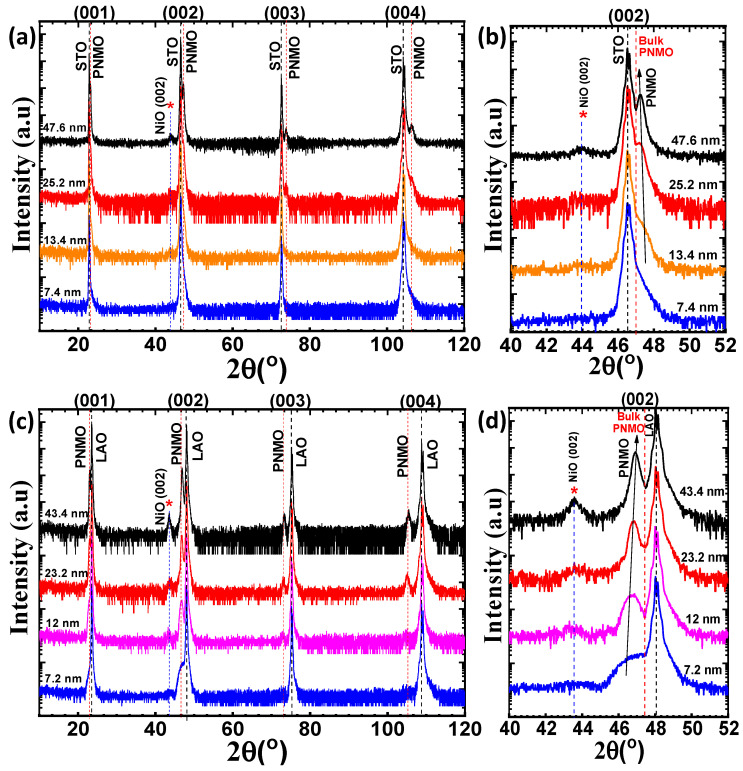
XRD θ/2θ scans of PNMO thin films grown/annealed at 800 °C under 350 mTorr O_2_ on (**a**) (001) STO and (**c**) (001) LAO substrates. (**b**) Zoom of the (002) reflection of both STO and PNMO. (**d**) Zoom of the (002) reflection of both LAO and PNMO. Parasitic phases are denoted by (*).

Figure 1a,c show the XRD diffraction patterns of the PNMO films deposited on STO (001) and LAO (001) substrates. Accordingly, the PNMO films (*a_p_*_PNMO bulk_*≈* 3.871 Å [29], where *a_p_* is the bulk pseudocubic cell parameter) grown on STO (*a_p_* _STO_ ≈ 3.905 Å) and LAO (*a_p_* _LAO_ ≈ 3.789 Å) substrates are under tensile strain and compressive strain, respectively. The highest intensity peak in each diffractogram corresponds to the STO and LAO substrate reflections (see dashed vertical black lines). Furthermore, all XRD patterns show a clear thickness dependence on structural properties in the PNMO films.

For more details, Figure 1b,d show the (002) reflection of both the STO and LAO substrates and the PNMO film, respectively. As expected, the (002) peak of the film is placed at a 2θ position larger (smaller) than that of the bulk PNMO (see dashed vertical red line) for the PNMO/STO (PNMO/LAO) substrate. This observation indicates that the out-of-plane *c* lattice parameter shrinks when the film is under in-plane tensile strain and expands when it is under in-plane compressive strain, in agreement with the lattice mismatch imposing a tensile and a compressive in-plane strain. At the same time, in Figure 1b,d, it can be appreciated that the position of the (002) peak shows a slight shift towards lower 2θ angles (higher 2θ angles) when increasing the film thickness for PNMO/STO (PNMO/LAO) substrate (see arrow). This indicates that the out-of-plane *c* lattice parameter of the film increases with increasing film thickness for STO, while it decreases for LAO, approaching the bulk value in both cases (see Figure 3a,b).

Finally, additional reflections denoted by (*), located at 2θ ≈ 43.9° (PNMO/STO films) and 2θ ≈ 43.5° (PNMO/LAO films), correspond to the parasitic NiO phase, as similarly observed in samples deposited at different pressures and temperatures [29]. The presence of the secondary NiO phase in the PNMO compound is not well understood. As the film thickness increases, the parasitic NiO peak increases in intensity. This fact points out the possibility of an increasing Pr-deficiency upon increasing the thickness, as revealed by EPMA for thick samples [29].

**Figure 2:**
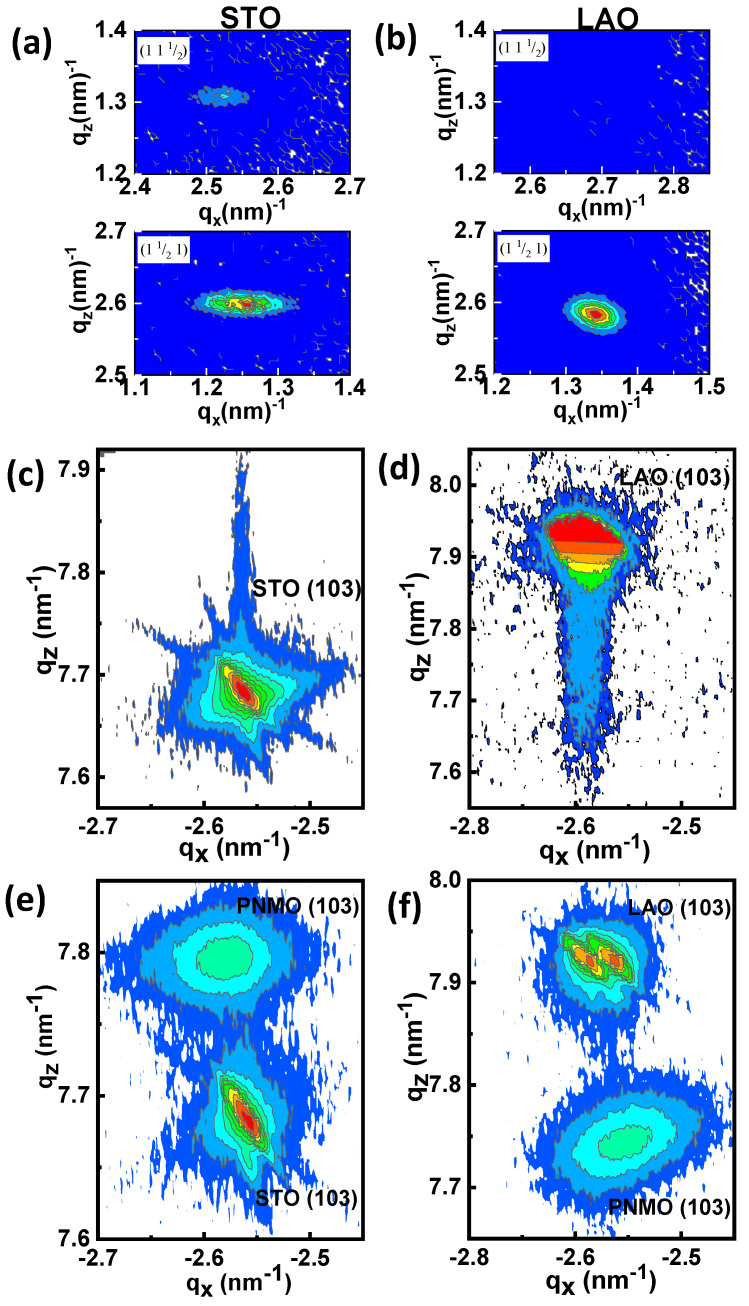
Integration along q_y_ of the intensity diffracted in the vicinity of the positions corresponding to (11 ½) and (1 ½ 1) for (**a**) the t = 47.6 nm PNMO/STO (001) film and (**b**) the t = 43.4 nm PNMO/LAO (001) film. Reciprocal Space Maps (RSMs) around (−103) reflections of PNMO films grown on STO with (**c**) t = 5.2 nm and (**e**) t = 47.6 nm and grown on LAO with (**d**) t = 4.7 nm and (**f**) t = 43.4 nm.

In order to discern the orientation of the film cell axes (monoclinic or orthorhombic) with respect to the substrate, we have explored, in reciprocal space (using a four circle diffractometer at the KMC-II beamline of the BESSY synchrotron), the appearance of the (021)_m_ reflection of PNMO (the subscript “m” stands for indexation using the monoclinic √2*a*_p_x√2*a*_p_x2*a*_p_ cell). This reflection is equivalent to (11½) of STO when *c* of PNMO is oriented along (001) of the substrate and is equivalent to (1½1) or (½11) of STO when *c* of PNMO lies along (010) or (100) of the substrate, respectively. As can be seen in Figure 2a, the two types of orientation are present for the 47.6-nm-thick PNMO film on STO (001) substrate, with a strong predominance of the orientation with *c* in-plane. On the contrary, in Figure 2b, for the 43.4-nm-thick PNMO film on LAO (001) substrate, the relative orientation of the monoclinic cell of the PNMO film is only oriented with *c* in-plane, and no domains with *c* out-of-plane can be detected.

In order to determine the values of the in-plane (*a*) and out-of-plane (*c*) lattice parameters, reciprocal space maps (RSMs) around (−103) reflection were performed on PNMO/STO and PNMO/LAO samples. In the RSMs shown in Figure 2c–f, the *x* axis corresponds to the in-plane q_x_ [100] direction, and the *y* axis corresponds to the out-of-plane q_z_ [001] direction. RSMs around the (−103) STO and (−103) PNMO reflections of the thinner (5.2 nm) and thicker (47.6 nm) PNMO/STO samples are shown in Figure 2c,e, respectively. Analog RSMs around the (−103) LAO and (−103) PNMO reflections of the thinner (4.7 nm) and thicker (43.4 nm) PNMO/LAO samples are shown in Figure 2d,f respectively. In Figure 2c,d, the RSMs for thinner films reveal both film and substrate (−103) diffraction spots are placed at the same position in q_x,_ so the estimated in-plane (*a*) pseudocubic cell parameters of the film coincide with those of the STO substrate (*a*_STO_ = 3.905 Å) and LAO substrate (*a*_LAO_ = 3.789 Å), showing that the films grow in-plane fully strained. On the contrary, the out-of-plane (*c*) lattice parameters were found to be *c* = 3.831 Å for PNMO/STO film and *c* = 3.890 Å for PNMO/LAO film, respectively.

Concerning the thicker films in Figure 2e,f, the RSMs reveal that the peak position q_x_ of (−103) PNMO film reflections is slightly shifted along the in-plane direction with respect to the position of the corresponding substrate, indicating a partial relaxation of the cell. This shift, in accordance with the strain induced, is towards larger absolute values of q_x_ for PNMO/STO and towards smaller absolute values for PNMO/LAO. From the positions of the peaks, the estimated cell parameters of a 47.6-nm thick PNMO/STO film are *a* = 3.877 Å and *c* = 3.848 Å, and those of a 43.4-nm thick PNMO/LAO film are *a* = 3.842 Å and *c* = 3.874 Å, respectively. These values are found to be similar to those of the La_2_NiMnO_6_ [6,36] and Pr_2_NiMnO_6_ systems [7].

**Figure 3:**
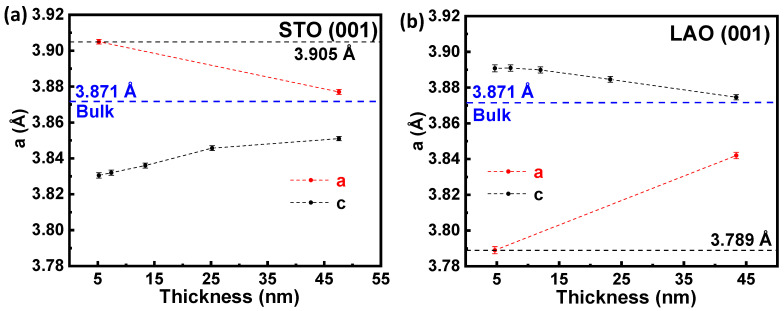
(**a**,**b**) variation of in-plane (red dashed line) and out-of-plane (black dashed line) lattice parameters of PNMO films as a function of thickness deposited on STO (001) and LAO (001) substrates. The blue and black dashed line represents the bulk counterpart value and the substrate lattice parameter.

The variation of the cell parameters (in pseudo-cubic notation) for both substrates is depicted in Figure 3a,b, respectively. It can be observed that when the film thickness increases, the in-plane (*a*) lattice parameter decreases (increases) for STO (LAO) towards the bulk value (see the red and blue dashed lines). Furthermore, the out-of-plane (*c*) lattice parameter progressively increases (decreases) for STO (LAO) with increasing thickness (see the black dashed line). In this regard, strain effects modify both in-plane and out-of-plane parameters by varying the film thickness. Both the tensile and compressive strains have a dominant effect in PNMO films with low thickness, affecting the lattice parameters strongly. Thus, the in-plane lattice parameters of the film tend to acquire the same value as that of the substrate. Therefore, from these observations, a (partial) relaxation of the in-plane (*a*) tensile strain and compressive strain takes place when film thickness increases, and consequently, the lattice parameters tend to acquire the bulk value (*a*_bulk_ = 3.871 Å) [37,38].

### 3.2. Magnetic Properties

**Figure 4:**
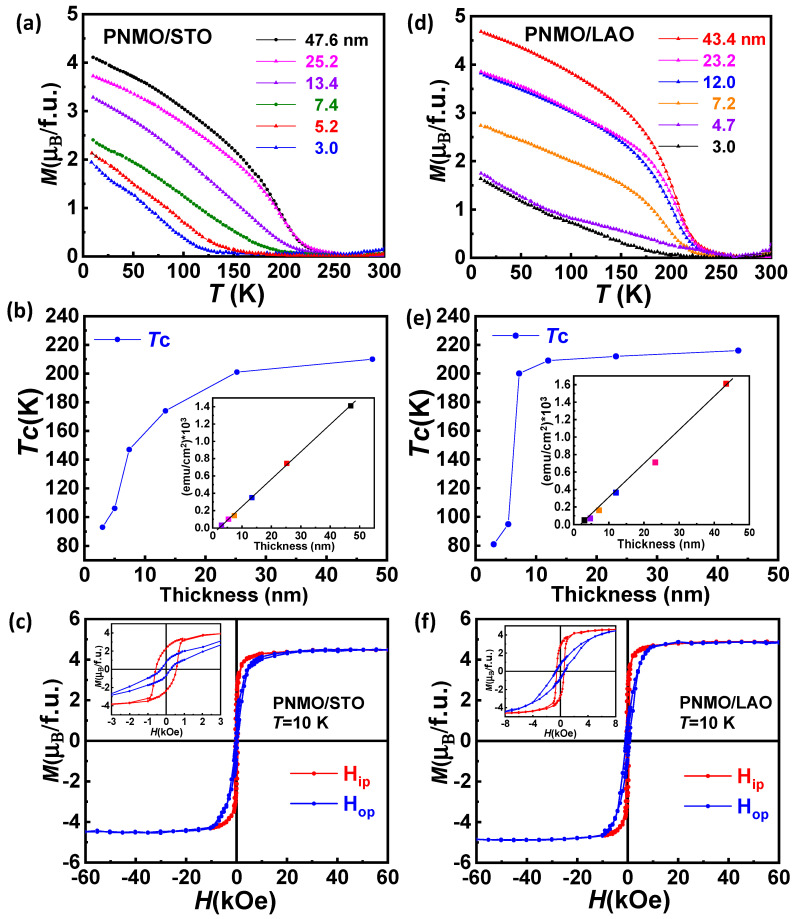
In-plane magnetization of (**a**) PNMO/STO and (**d**) PNMO/LAO thin films of different thicknesses as a function of temperature under an applied magnetic field of µ_0_H = 0.5 T. Curie temperatures T_C_ of (**b**) PNMO/STO and (**e**) PNMO/LAO thin films as a function of thickness. Insets of (**b**,**e**) show the magnetization (emu/cm^2^)*10^3^ at 10 K as a function of film thickness. M(H) hysteresis loops (measured at 10 K) with in-plane (red curve) and out-of-plane (blue curve) applied magnetic fields of (**c**) 47.6-nm-thick PNMO film deposited on STO substrate and (**f**) 43.4-nm-thick PNMO film deposited on LAO substrate. Insets (**c**,**f**) show the low field region in detail.

In order to explore the thickness dependence on the magnetic properties, Figure 4 shows the in-plane magnetization of PNMO/STO and PNMO/LAO samples of different thicknesses (t) as a function of temperature under an applied magnetic field of 5 kOe. Temperature-dependent magnetization *M*(*T*) of PNMO films grown on STO and LAO substrates with different thicknesses is depicted in Figure 4a,d, respectively. The T_C_ value (estimated from the inflection point) was extracted and plotted in Figure 4b,e. From the results, it can be appreciated that the magnetization and the Curie temperature T_C_ (onset of the ferromagnetic behavior) reach lower values as the film thickness decreases. Therefore, a notable degradation of the magnetic properties takes place as the samples become thinner.

In fact, the absence of a ferromagnetic ordering has been reported in ultrathin films (t < 4 nm) [39,40,41]. This could be attributed, as a first approximation, to the existence of an interfacial dead layer that modifies the magnetic and structural properties. Some factors that contribute to the formation of a dead layer effect on very thin films could be a chemically and/or structurally altered film-substrate interface as well as a discontinuous film coverage over the substrate surface during the initial film growth [42]. The insets in Figure 4b,e depict the magnetization (emu/cm^2^)*10^3^ at 10 K as a function of thickness. Therefore, by extrapolating to zero, the thickness of the dead layer for PNMO films was estimated to be around ~3 nm (on both substrates).

At the same time, it should also be noticed that the M(T) curve, for the thickest (47.6 nm) PNMO/STO film, displays a FM transition at T_C_ ≈ 210 K and a saturation magnetization of M_s_ ≈ 4.5 µ_B_/f.u. at 10 K (see Figure 4c), which is very similar to that reported in the literature and close to the bulk value [7]. The thickest (43.4 nm) PNMO/LAO film displays T_C_ ≈ 216 K and M_s_ ≈ 4.85 µ_B_/f.u. at 10 K (see Figure 4f), also very close to the bulk value *M*_s_ = 5 µ_B_/f.u. [6]. For comparison, the Curie temperature (T_C_), saturation magnetization (M_s_), coercive field (H_C_), and remanence magnetization (Mr) data are listed in Table 1 for thicker samples (on both substrates). In this regard, the M(H) curves reveal that the easy magnetization axis prevails in the IP orientation for both substrates. The H_C_ and Mr reinforce that the easy axis lies in the IP orientation. On the other hand, a coercive field H_C_ of about 565 Oe (IP field) and 264 Oe (OP field) is found for the 47.6-nm-thick PNMO/STO film, while a coercive field H_C_ of about 538 Oe (IP field) and 631 Oe (OP field) is found for the 43.4-nm-thick PNMO/LAO film. This could indicate that the IP anisotropy is higher in the PNMO/LAO film, in agreement with the larger coercive field (OP), than in the PNMO/STO film.

### 3.3. XAS and XMCD

To evaluate the spin and orbital moments, the valence state of ions, and the nature of the ferromagnetic (FM) interactions in the PNMO compounds, XAS and XMCD measurements were carried out at the Ni *L*_2,3_, Mn *L*_2,3_, Pr *M*_4,5_, and O *K* edges. At the same time, in order to qualitatively analyze the ferromagnetic character of the PNMO system, we performed X-ray spectroscopic calculations using CTM4XAS [43] and Crispy software [44,45]. For this study, we have chosen three PNMO samples of different thicknesses and different Curie temperatures (high-*T*_C_ and low-*T*_C_) deposited on STO and LAO substrates. The first two samples with good FM properties were a 47.6-nm-thick PNMO/STO sample (T_C_ ≈ 210 K and Ms ≈ 4.5 µ_B_/f.u at 10 K) and another 43.4-nm-thick PNMO/LAO sample (T_C_ ≈ 216 K and Ms ≈ 4.7 µ_B_/f.u at 10 K) with a Pr:(Ni + Mn) ratio of ~0.86 (Pr_1.7_Ni_0.9_Mn_1.1_O_6-y_) [29]. The third was a 4.7-nm-thick PNMO/LAO sample with poor FM properties (T_C_ ≈ 95 K). In the following, the PNMO samples grown on STO substrate (high-*T*_C_) were labeled as PNMO/STO-(A), and the thicker (high-*T*_C_) and thinner (low-*T*_C_) samples grown on LAO substrates were labeled as PNMO/LAO-(B) and PNMO/LAO-(C), respectively.

#### 3.3.1. Ni and Mn L_2,3_ Edges

**Figure 5:**
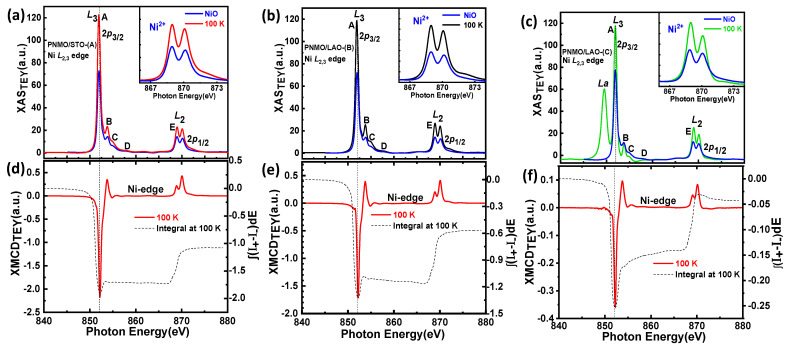
Experimental XAS spectra at Ni-*L*_2,3_ edges for (**a**) PNMO/STO-(A), (**b**) PNMO/LAO-(B), and (**c**) PNMO/LAO-(C). The NiO XAS (blue curve) is also plotted for comparison. The corresponding XMCD signals at 100 K are plotted in (**d**–**f**) panels. Dashed black lines show the XMCD integral.

Considering that the ferromagnetic character of Pr_2_NiMnO_6_ is explained in terms of the superexchange interactions between Ni^2+^ and Mn^4+^ according to the Goodenough-Kanamori rules, it is of major interest to determine the valence states and orbital occupancies of these two ions. For this purpose, we recorded the XAS and XMCD spectra across the Ni-*L*_2,3_ edges (2*p*→3d transitions) for the three samples mentioned earlier (see Figure 5a–c).

We found that the strong Ni *L*_3_ edge peak and the Ni *L*_2_ edge double-peak structure of (a) PNMO/STO-(A), (b) PNMO/LAO-(B), and (c) PNMO/LAO-(C) samples were very similar to those already reported for La_2_NiMnO_6_ [46,47], Pr_2_NiMnO_6_ [28], and Nd_2_NiMnO_6_ [48] double perovskites. For the thinner PNMO/LAO-(C) sample (low-*T*_C_), a sharp peak appears at around 849.5 eV, which is due to the La *M*_4_ (3*d*_3/2_→4*f*) XAS from the substrate. Beyond this particularity, we can say that both the Ni *L*_3_ (*ħυ* ≈ 850 − 855 eV) and *L*_2_ edge (ℏυ ≈ 865 − 875 eV) XAS line shapes are quite similar and can be easily compared with the corresponding spectra of other divalent Ni^2+^ compounds, such as isoelectronic NiO (also shown in the inset) or Ni dihalides [49].

According to the electric dipole selection rules, Ni 2*p* electrons may be excited into empty states either with 3*d* or 4*s* symmetry. The 2*p*→3*d* transitions are about 30 times stronger in intensity than 2*p*→4*s* ones due to the large overlap of the 3*d* wave functions with the 2*p* ones (Fermi’s golden rule) [50]. The presence of this double-peak structure (labeled as E and shown in the inset) in Ni *L*_2_ edges is nevertheless well understood in terms of a covalent ground state of mainly Ni^2+^ (3*d*^8^) character, which in O*_h_* symmetry can be written as ^3^A_2g_ ($t_{2g}^{6}\;e_{g}^{2}$) plus an anion-dependent fraction of the 3*d*^9^*L* and 3*d*^10^*L*^2^ configurations, where *L* corresponds to a ligand hole in the O 2*p* state [49]. This double-peak at the Ni *L*_2_ edge was previously observed in a nonstoichiometric sample of the La_2_Ni_1-x_Mn_1+x_O_6_ series [51]. In a similar way, this double peak was also found in stoichiometric samples of *R*_2_NiMnO_6_ (*R* = La, Pr, and Nd) with almost full cationic ordering, yet very different from the *L*_2_ peak of PrNiO_3_ and NdNiO_3_ corresponding to Ni^3+^ [28,46,48]. Therefore, we can conclude that Ni ions in our three PNMO samples are in a divalent state with a high-spin (HS) electronic configuration (Ni^2+^:$t_{2g}^{3}↑t_{2g}^{3}↓e_{g}^{2}↑$) [52,53]. We further examined this point by means of XAS and XMCD simulations (see Section 3.3.2).

**Figure 6:**
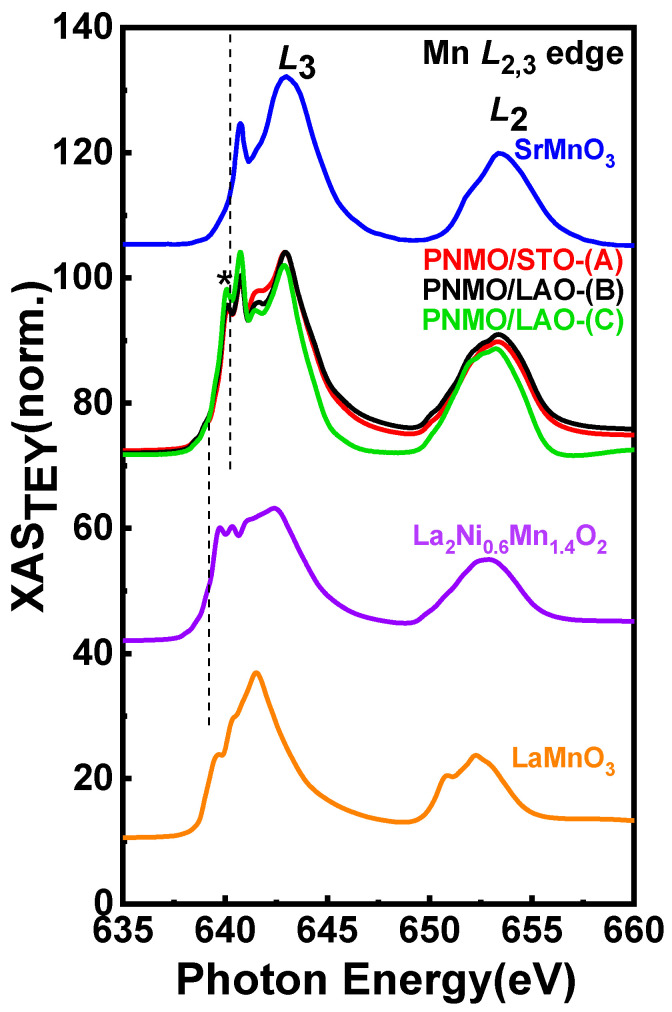
XAS spectra across the Mn *L*_2,3_ edges for PNMO/STO-(A), PNMO/LAO-(B), and PNMO/LAO-(C) samples. LaMnO_3_, La_2_Ni_0.6_Mn_1.4_O_6_ and SrMnO_3_ XAS are shown for comparison.

**Figure 7:**
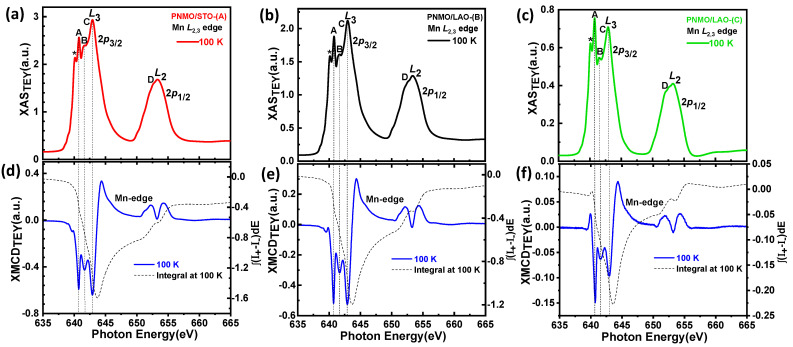
Experimental Mn-*L*_2,3_ XAS spectra for (**a**) PNMO/STO-(A), (**b**) PNMO/LAO-(B), and (**c**) PNMO/LAO-(C) samples at 100 K. The respective XMCD signals are plotted in the (**d**–**f**) panels, where the dashed black line shows the corresponding XMCD integrals.

The Mn-*L*_2,3_ edge XAS spectra as collected by TEY at T = 100 K for the three PNMO samples investigated are shown in Figure 6 and Figure 7. They all look very similar to the XAS previously reported for La_2_NiMnO_6_ [46,47], Pr_2_NiMnO_6_ [28], and Nd_2_NiMnO_6_ [48] double perovskites. Additionally, in order to qualitatively evaluate the valence state of Mn ions in our three PNMO samples, we have also recorded the Mn *L*_2,3_ XAS of some reference samples: LaMnO_3_ (Mn^3+^), La_2_Ni_0.6_Mn_1.4_O_6_ (mixed-valence, Mn^3.6+^) and SrMnO_3_ (Mn^4+^) (see Figure 6). The energy position (particularly at the *L*_3_-edge) and the overall spectral shape of our PNMO samples are quite similar to those of SrMnO_3_ and to other nominal Mn^4+^ references with O*_h_* crystal field symmetry, like LaMn_0.5_Ni_0.5_O_3_ [54], LaMn_0.5_Co_0.5_O_3_ [55], and Ca_3_CoMnO_6_ [56], but clearly different from those in LaMnO_3._ Nevertheless, a small feature at approximately 640.2 eV, labeled with (*), and identified as due to Mn^2+^ is also present in our case. This could be related to surface contamination in the films. In any case, the overall Mn-*L*_2,3_ XAS spectra of PNMO and SrMnO_3_ are quite similar, which indicates that Mn in our PNMO samples is very likely, mostly in a tetravalent state (Mn^4+^:$t_{2g}^{3}↑$) [52,53]. In order to confirm this hypothesis, as in the case of the Ni-*L*_2,3_ edges, we performed XAS and XMCD calculations, which are shown in Section 3.3.2.

In Figure 7a–c, the Mn *L*_3_-edge main x-ray spectroscopic structures have been labeled as A, B, and C, while D corresponds to the *L*_2_-edge. In addition, the presence of Mn^2+^ is denoted by (*). All three samples show similar Mn-*L*_2,3_ XAS (see Figure 6), but the D feature in the PNMO/LAO-(C) sample is slightly more prominent than in the other two samples.

At the same time, it also shows slight differences in the intensity of peaks A, B, and C. In this case, the branching ratio (defined as I(*L*_2_)/I(*L*_3_), where I(*L*_3_) and I(*L*_2_) are the XAS maximum amplitudes at the *L*_3_ and *L_2_* peaks, respectively) is larger for the PNMO/LAO-(C) sample (=0.581). This might be associated with an electron-yield saturation effect at the Mn *L_3_*-edge due to the thickness of the samples [57,58,59,60]. With PNMO/LAO-(C) being the thinnest sample (4.7 nm), a saturation effect would enhance the intensity of the spectroscopic features at low energy within a given absorption edge as compared to those in the higher energy part. Saturation effects result in a recorded signal that is not proportional to the photoabsorption cross-section as the photon energy is varied. In this case, the intensities of prominent absorption peaks get reduced or “saturated” [61]. Though being stronger at more grazing photon incidence, in sufficiently thin films saturation can also affect spectra recorded at normal incidence [62].

X-ray magnetic circular dichroism (XMCD) was used to investigate the specific magnetic ordering, namely the nature of exchange couplings between the different magnetic sites (Ni, Mn, and Pr) and O in the PNMO compounds. Panels (d), (e), and (f) of Figure 5 and Figure 7 show the Ni and Mn *L*_2*,*3_ edge XMCD spectra for PNMO/STO-(A), PNMO/LAO-(B), and PNMO/LAO-(C) films, as recorded at T = 100 K, under an applied field of 2 T. This temperature value was chosen to lie well below the high-temperature magnetic transition at 216 K. We note that all XMCD spectra were normalized to the integrated area of the corresponding XAS spectra to ease their comparison [53,54]. Looking at the large negative XMCD signal in both the Mn and Ni-*L*_3_ regions, we can extract that the Mn and Ni spin moments are ferromagnetically coupled to each other, as also proposed in La_2_NiMnO_6_ [46].

In order to extract quantitative information about the orbital angular *µ*_orb_ and spin magnetic moment *µ*_spin_ contributions to Mn 3*d* and Ni 3*d* state magnetization, we applied the sum-rules to the XMCD spectra. For this, we took the threshold between the 2*p*_3*/*2_ and 2*p*_1*/*2_ regions at 650 eV for the Mn *L*_2*,*3_ edges and at 865 eV for the Ni *L*_2*,*3_ edges and neglected the contribution of the magnetic dipole operator T_Z_ [46,63,64]. We have that:

(1) $$\mu_{L}=-\frac{4\left(\int_{L_{3}}^{}\Delta I\left(E\right)dE+\int_{L_{2}}^{}\Delta I\left(E\right)dE\right)}{3\left(\int_{L_{3}}^{}I\left(E\right)dE+\int_{L_{2}}^{}I\left(E\right)dE\right)}\left(10-N_{3d}\right)$$

(2) $$\mu_{S}+7\mu_{T}=-\frac{2\int_{L_{3}}^{}\Delta I\left(E\right)dE-4\int_{L_{2}}^{}\Delta I\left(E\right)dE}{\int_{L_{3}}^{}I\left(E\right)dE+\int_{L_{2}}^{}I\left(E\right)dE}\left(10-N_{3d}\right)$$

where Δ*I* = *I*^+^ − *I*^−^; *I* = *I*^+^ + *I*^−^; *N*_3d_ is the 3*d* electron occupation number; and *µ_T_* is the magnetic dipole moment (usually negligible for transition metals in a local octahedral environment). The corresponding integral of the XMCD signal is also depicted in panels (d), (e), and (f) of Figure 5 and Figure 7.

Therefore, using Equations (1) and (2), at 100 K, we obtained $\mu_{L}^{Ni}/\mu_{S}^{Ni}=0.24$1 and $\mu_{L}^{Mn}/\mu_{S}^{Mn}=0.099$ for PNMO/STO-(A), $\mu_{L}^{Ni}/\mu_{S}^{Ni}=0.167\;$and $\mu_{L}^{Mn}/\mu_{S}^{Mn}=0.055$ for the PNMO/LAO-(B) sample, and for the thinner PNMO/LAO-(C) sample (low-T_C_), we obtained $\mu_{L}^{Ni}/\mu_{S}^{Ni}=0.077$ and $\mu_{L}^{Mn}/\mu_{S}^{Mn}=-0.018$. These values are similar to those in previous reports [46,54]. The orbital moment values we obtained are in all cases compatible with Mn^4+^ ions, where they are expected to be quenched. Regarding the spin moment, we must note that the difficulty in separating the L_3_ from the L_2_ edges of Mn^4+^ introduces a large degree of uncertainty. Following [65,66], μ_S_ could be underestimated by a factor of 0.59. In the case of Ni ions, the spin-orbit coupling is larger, and the spin momentum calculated value using the corresponding XMCD-derived sum rule is estimated not to deviate more than 10% from the actual value [67].

Then, based on the XMCD data, we can say that (i) the Mn orbital to spin moment ratio is directly proportional to thickness, being nearly quenched in the thinnest sample investigated, and (ii) the Ni XMCD signal being in general very small at the *L*_2_ edge while still largely negative at the *L*_3_ edge indicates a very large orbital contribution to the Ni magnetic moment. This allows us to conclude that the magnetic anisotropy observed in the magnetic measurements (M(H) loops of the PNMO/STO-(A) and PNMO/LAO-(B) samples) is of magnetocrystalline origin and induced by Ni (see Figure 4c,f).

#### 3.3.2. Numerical Simulation of XAS and XMCD Spectra of Ni^2+^ and Mn^4+^ Edges

**Figure 8:**
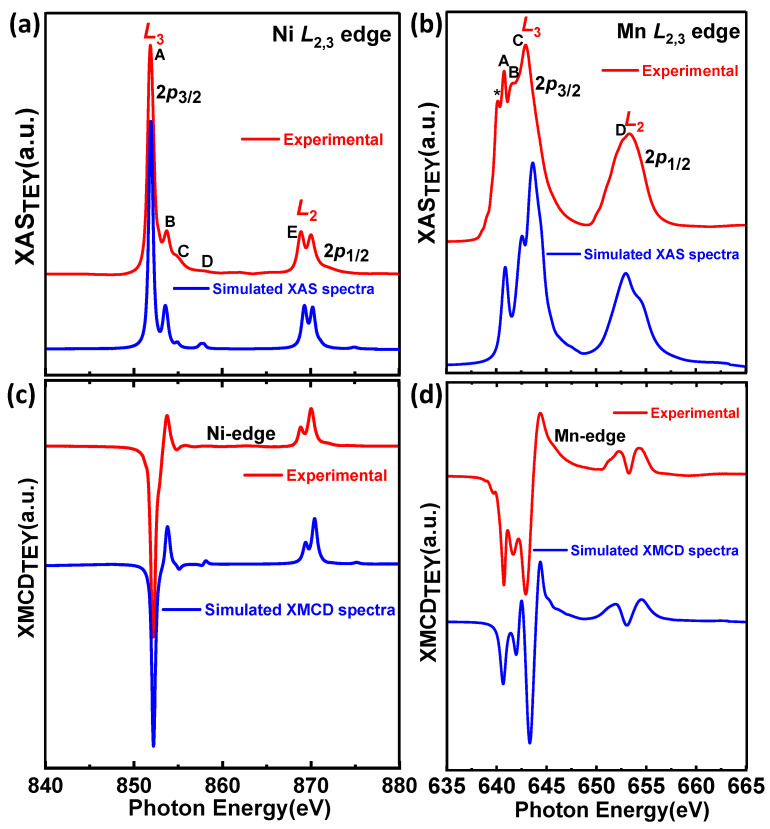
Calculated and experimental XAS (**a**,**b**) and XMCD (**c**,**d**) across Ni (**a**,**c**) and Mn (**b**,**d**) *L*_2,3_ edges (*O*_h_ symmetry, blue lines). The experimental spectra of the PNMO/STO-(A) sample (red lines) are shown for comparison.

In order to obtain more detailed information on the local electronic structure of Ni and Mn edges of our PNMO compounds, theoretical simulations of the XAS and XMCD spectra were performed to fit the experimental data using CTM4XAS [43]. Figure 8 displays the best calculated XAS and the corresponding XMCD spectra for Ni^2+^ and Mn^4+^ ions. For the sake of comparison, the experimental spectra of the PNMO/STO-(A) sample are also plotted.

The Ni and Mn-*L*_2,3_ edges spectra are calculated from the sum of all possible transitions for an electron excited from the 2*p* core level to an unoccupied 3*d* level. The ground state is approximated by the electronic configuration 3*d*^n^. For a transition-metal ion in octahedral symmetry, the crystal field multiplet calculation uses an empirical value of the crystal field splitting 10 D*q* (energy between the t_2g_ and e_g_ states). In the ground state, both the 3*d* spin−orbit coupling and the crystal field 10 D*q* affect the 3*d*^n^ configuration. The 3*d*^n^ ground state and the 2p^5^3*d*^n+1^ final state are affected by 3*d*3*d* and 2*p*3*d* intra-atomic Coulomb interactions (*U_dd_*, *U_pd_*). The 2*p* and 3*d* spin−orbit couplings and local crystal field, which are described with empirical parameters (10 Dq, Ds, Dt, and M) in appropriate symmetry, are also included [68,69,70]. In addition, the charge-transfer energy ∆ (needed to transfer one electron from the ligand band to the transition-metal site) is strongly anion dependent, being given roughly by the electronegativity difference between anion and cation. For high covalency, ∆ may be in the negative regime due to the strong hybridization with the oxygen band [71].

In the PNMO compound, Ni^2+^ ions are surrounded by oxygen octahedra, and their ground state ionic configuration (as a first approach) in O*_h_* symmetry can be written as ^3^$A_{2g}\left(t_{2g}^{6}e_{g}^{2}\right)$. On the other hand, Mn^4+^ ion valence band filling can be written as ^4^$A_{2g}\left(t_{2g}^{3}\right)$, also in O*_h_* symmetry [28]. For Ni^2+^ XAS calculations (see Figure 8a,c), *d*^9^*L* and *d*^10^*L*^2^ had to be actually considered, where *L* corresponds to a ligand hole in the O 2*p* state. As in the case of Mn^4+^ ions (see Figure 8b,d), this is due to the large covalency of metal-oxygen bonds, which renders the ionic approximation very inaccurate. In this latter case, the ground state could be well described using *d*^3^ and *d*^4^*L* configurations [72].

In Figure 8a,c, when dealing with Ni-*L*_2,3_XAS and XMCD calculations, we observed that the double peak feature at both the *L*_3_ and *L*_2_ edges gets strongly affected by the charge transfer energy parameter (∆). So, for small (<3 eV) or even negative ∆ values, calculations do not properly fit the experimental data at the *L*_2_ edge, which leads to the formation of weak satellites and to changes in the multiplet structure. A good fit requires using a moderately positive ∆ value (∆ = 3.0 eV) and a crystal-field energy of 10Dq = 1.2 eV (see more details in Table 2). On the other hand, the number of holes for the Ni-*L*_2,3_ XAS and XMCD calculations is 1.82, which is in good agreement with the ionic expected value of Ni^2+^ (3*d*^8^ configuration plus some contribution of 3*d*^9^*L* and 3*d*^10^*L*^2^).

Figure 8b,d display the calculated XAS and XMCD spectra for Mn^4+^ ions for the PNMO compound. By comparing the XAS and XMCD spectra (experimental and simulated), it can be observed that the multiplet structure (spectrum shape) and the peaks marked as A, B, C, and D at the Mn-*L*_3_ and Mn-*L*_2_ edges fit well with the experimental data. For that, we used 10Dq = 2.5 eV and ∆ = 2.5 eV (see more details in Table 2). The number of holes that follows from these calculations is 6.69 per Mn atom.

Therefore, according to these results, we can conclude that Ni and Mn cations, respectively, adopt dominant divalent and tetravalent oxidation states in O*_h_* local symmetry and HS configuration. On the other hand, considering that FM ordering in the PNMO double perovskite structure is due to the Ni^2+^–O– Mn^4+^ superexchange interactions according to the Goodenough-Kanamori rules, XAS measurements at the Ni and Mn *L*_2,3_ edges in our PNMO compound allow us to confirm this statement even in samples with low-*T*_C_. Similar results were observed on LNMO compounds [73].

#### 3.3.3. Pr M_4,5_ Edges

**Figure 9:**
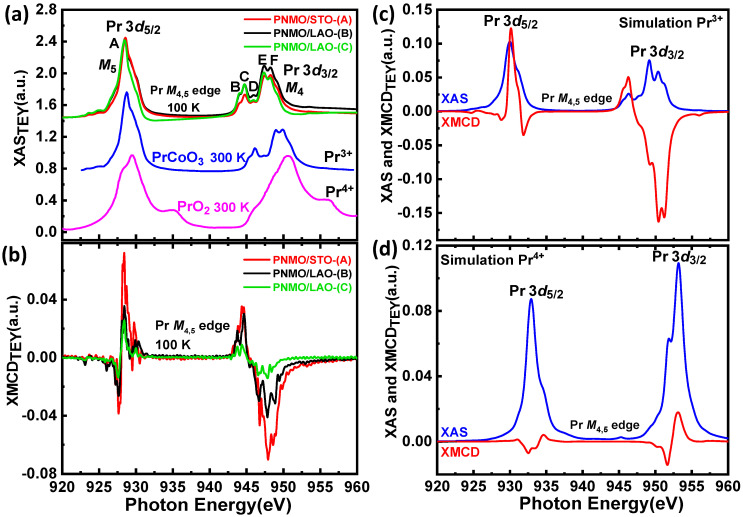
(**a**) Pr *M*_4,5_ XAS for PNMO/STO-(A), PNMO/LAO-(B), and PNMO/LAO-(C) as collected at T = 100 K. For comparison, the XAS spectra of PrCoO_3_ (Pr^3+^) and PrO_2_ (Pr^4+^) at T = 300 K are also shown [74,75,76]. (**b**) Corresponding XMCD spectra under 2 T. (**c**,**d**) Calculated Pr *M*_4,5_ XAS and XMCD spectra for Pr^3+^ and Pr^4+^ isolated cations, respectively.

The Pr *M*_4,5_ XAS spectra for the three samples under study with very different *T*_C_ values, which probe the unoccupied density of 4*f* states, are shown in Figure 9a. The spectra of PrCoO_3_ (Pr^3+^) and PrO_2_ (Pr^4+^) at T = 300 K are used as references [74,75,76] in the same plot. In the literature, the experimental XAS spectra of formally tetravalent 4*f* oxides (namely PrO_2_, CeO_2_, and LaO_2_) show a main broad peak at both the M_5_ and M_4_ absorption edges [75,76]. Calculations for PrO_2_ in ref [75] finely reproduced its spectroscopic structure. In contrast, the richer multiplet structure of Pr^3+^-based bands is determined by a strong Coulomb interaction between the two 4*f* electrons as well as by a covalent mixing with oxygen 2*p* states. This has been earlier shown by XAS calculations for La^3+^, Ce^3+^ and Pr^3+^-based compounds [77]. In the case of our Pr *M*_4,5_ XAS spectra taken from PNMO samples, we can see in Figure 9a that it strongly resembles that of PrNiO_3_ [77] and PrCoO_3_, pointing to a trivalent state of Pr cations.

Nevertheless, in order to confirm this statement, we performed XAS and XMCD calculations using the Crispy interface, based on the Quanty code [44,45]. We considered both Pr^3+^ and Pr^4+^ ions, where the dipolar electronic transitions are 3*d*^10^4f^2^→3*d*^9^4f^3^ and 3*d*^10^4f^1^→3*d*^9^4f^2^, respectively, and O*_h_* crystal field symmetry (see Figure 9c,d). Only the Pr^3+^ calculations were able to properly reproduce all XAS features (spectral shape and amplitude). We can thus conclude that the A-site deficiency in our Pr_2−δ_Ni_1−x_Mn_1+x_O_6−y_ compound, as analyzed by EPMA [29], has no evident impact on the oxidation state of Pr.

Regarding the XMCD, the experimental results obtained at Pr *M*_4,5_ edges in our three samples (see Figure 9b) show a dichroic signal with the opposite sign as compared to that found at the Ni and Mn *L*_2,3_ edges. In the case of rare earths, the final state multiplet 3d^10^4f^N^ → 3d^9^4f^N+1^ is split in two parts by the 3d spin-orbit interaction, and, depending on *N*, the description involves the discrete energy levels of the initial and final state N-particle wavefunctions (multiplets) [78]. Each multiplet state has a definite atomic angular momentum quantum number *J*. In this description, the dipole selection rules are ∆*J* = 0, ±1, and only a part of these lines can be reached from the initial state [77,79].

At the same time, XMCD involves the contributions of dipole transitions with the effects of interatomic hybridization between the 4f states of Pr and the 3d states of Mn and Ni, and intra-atomic exchange interaction between the 4f–5d states of Pr. This implies that the inverted XMCD signal could be related to the coupling of the 4f electrons with the valence band as well as 3d–4f electron-electron interactions [78]. Moreover, there is a strong correlation between 3d→4f transitions (∆*J* = −1) of the Pr *M*_4,5_ edge. Hence, the ∆*J* = −1 terms could also be dominating in the XMCD spectrum, giving rise to an inverted dichroic signal. Otherwise said, this is not due to an antiparallel alignment of the Pr moments to the externally applied magnetic field or to Ni and Mn spins.

Concerning the calculated XMCD spectra, the Pr^3+^ case fits pretty well with the experimental data, which further corroborates that the valence state of Pr in our samples is 3+.

#### 3.3.4. O K Edge

**Figure 10:**
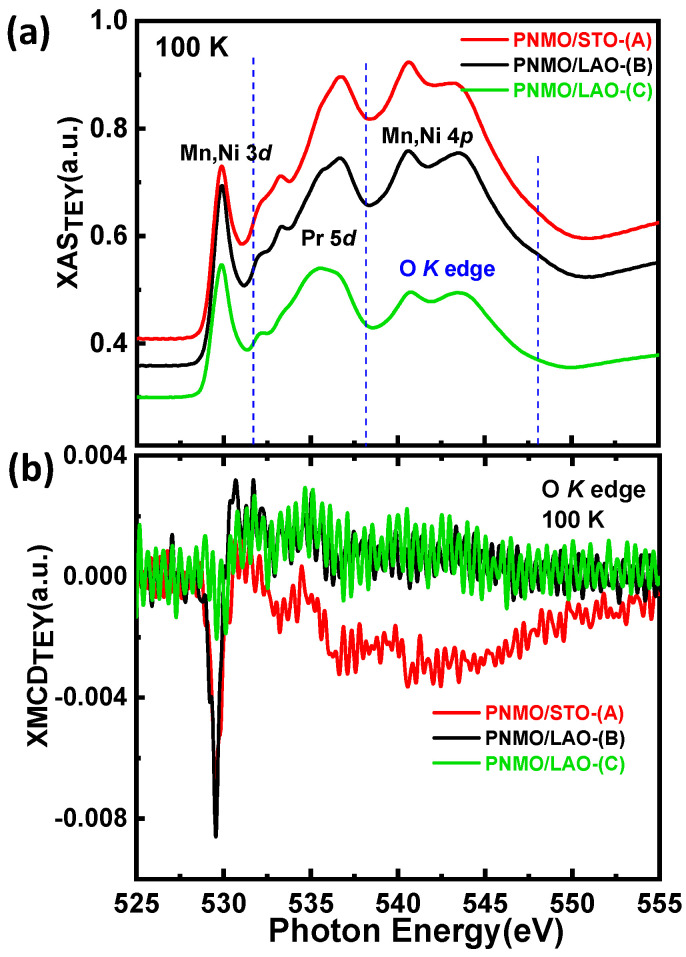
(**a**) O *K*-edge XAS spectra for the same three samples collected at T = 100 K. (**b**) XMCD data collected over O *K*-edge at T = 100 K under an applied field of 2 T.

Figure 10a shows the O *K* XAS edge spectra of the same three samples with high-*T*_C_ and low-*T*c measured at T = 100 K in TEY mode. Focusing on the pre-edge zone, we can analyze the hybridization of oxygen valence states with unoccupied Ni/Mn 3*d* and Pr 5*d* bands [80]. The first peak found, located around 529.8 eV (in the three samples), corresponds to available O 2*p*–Ni/Mn 3*d* states. At higher energies, the broad structure around 535 eV corresponds to O 2p mixing with Pr 5*d* states, and the bumps around 540–545 eV are due to the hybridization of O 2*p* with Ni and Mn 4*sp* bands, which are consistent with earlier results reported on LaMnO_3_, LaFeO_3_, and LaCoO_3_ [81,82,83].

The XMCD results obtained at the O *K*-edge in our three samples are shown in Figure 10b. These are particularly relevant since the magnetic interaction is mediated by O ions. In Figure 10b, one can observe a strong negative peak that is more prominent for samples PNMO/STO-(A) and PNMO/LAO-(B), with an intensity equivalent to several percent of the total XAS intensity of the t_2g_ region. This O *K*-edge XMCD signal is attributed to the 3*d* orbital moment on the neighboring sites of the Ni or Mn ions interacting through the *p*-*d* hybridization [84]. The intensity of this peak becomes much lower for the PNMO/LAO-(C) sample, which corroborates the loss of ferromagnetism in this last sample. On the other hand, because of the absence of spin-orbit splitting for the 1s core level, the O *K*-edge XMCD spectra show the orbital moment simply but are insensitive to the spin moment. Therefore, the integral area of the O *K*-edge XMCD is directly proportional to the orbital moment, and, since the negative XMCD signal indicates a positive magnetic moment (*µ*_L_ > 0), the orbital magnetic moment of O 2*p* is parallel to that of Ni/Mn 3*d* [85,86].

## 4. Conclusions

In summary, epitaxial PNMO thin films with varying thickness have been prepared on (001) STO and (001) LAO substrates under optimized growth conditions (grown/annealed at 800 °C under 350 mTorr O_2_) by the RF sputtering technique. PNMO films show a strong dependence of structural and magnetic properties on film thickness. Particularly, reciprocal space maps (RSMs) around (−103) reflection (on both substrates) reveal a (partial) relaxation of the in-plane tensile strain and compressive strain when film thickness increases; therefore, in-plane lattice parameters approach the bulk value. As structural strain decreases with increasing film thickness, the ferromagnetic behavior (on both substrates) improves and is optimal for the thicker PNMO films. In this regard, the Curie temperature (*T*_C_) and saturation magnetization (*M*s) (i.e., for 47.6-nm-thick PNMO/STO film, T_C_ ≈ 210 K and Ms ≈ 4.5 µ_B_/f.u. and, for 43.4-nm-thick PNMO/LAO film, T_C_ ≈ 216 K and Ms ≈ 4.85 µ_B_/f.u., at 10 K) display values very close to the bulk value. In fact, M(H) curves reveal that the IP orientation of the easy magnetization axis prevails. Consequently, for thinner films, lattice distortion, oxygen deficiency, and sample inhomogeneity could induce a loss of ferromagnetism at the film-substrate interface. XAS and XMCD measurements on some characteristic samples (high and low-*T*_C_) deposited on STO and LAO substrates reveal that irrespective of the structural strain state (tensile or compressive) and the film thickness, the oxidation states of Ni and Mn ions are stabilized as Ni^2+^ and Mn^4+^, even in samples with poor magnetic properties. In addition, based on the XMCD data, the very large orbital moment contribution to the magnetic moment of Ni ions (on both substrates) allows us to conclude that the magnetic anisotropy observed in the magnetic measurements (M(H) loops) is of magnetocrystalline origin induced by Ni. At the same time, the Pr *M*_4,5_ edge XAS spectra of the rare earth element reveal that the valence state of the Pr ions is 3+, indicating that the Pr deficiency in our Pr_2−δ_Ni_1−x_Mn_1+ x_O_6−y_ (PNMO) compound (as analyzed by EPMA) had no evident impact on the oxidation state of Pr. Theoretical simulations based on a charge transfer multiplet model of XAS and XMCD data at Ni *L*_2,3_ and Mn *L*_2,3_ edges allow us to conclude that the experimental spectra are in good agreement with the calculated spectra of Ni^2+^ and Mn^4+^ in O*_h_* symmetry and high-spin configuration.

## Figures and Tables

**Table 1:** Magnetic properties of 47.6-nm-thick PNMO/STO film and 43.4-nm-thick PNMO/LAO film.

M(H) loops	T_C_(K)	Ms(µ_B_/f.u)	Hc(Oe)	Mr (µ_B_/f.u)
47.6 nm-PNMO/STO film at 10 K				
ip	210	4.5	565	2.3
op			264	0.8
43.4 nm-PNMO/LAO film at 10 K				
ip	216	4.85	538	2.8
op			631	0.7

**Table 2:** Best-fit parameters used for the multiplet calculations-based simulations of the experimental Ni and Mn *L*_2,3_- edge XAS and XMCD spectra of PNMO systems.

Parameter	Ni^2+^	Mn^4+^
Site symmetry	O*_h_*	O*_h_*
Crystal Field (*10Dq*) (eV)	1.2	2.5
Charge transfer energy (∆) (eV)	3.0	2.5
U_dd_(eV)	7.5	6.5
U_pd_(eV)	7.3	8.5
Slater’s integrals reduction (%) (F_dd_, F_pd_, G_pd_)	0.8	0.7
Majority state	*d* ^8^	*d* ^3^
Minority state	*d* ^9^ *L*	*d* ^4^ *L*

## Data Availability

Not applicable.
